# Validation of the new EPIC DNA methylation microarray (900K EPIC v2) for high-throughput profiling of the human DNA methylome

**DOI:** 10.1080/15592294.2023.2185742

**Published:** 2023-03-05

**Authors:** Aleix Noguera-Castells, Carlos A. García-Prieto, Damiana Álvarez-Errico, Manel Esteller

**Affiliations:** a Cancer Epigenetics Group, Josep Carreras Leukaemia Research Institute (IJC), Barcelona, Spain; b Department of Biosciences, Faculty of Science, Technology and Engineering, University of Vic - Central University of Catalonia (UVic-UCC), Vic, Spain; c Life Sciences Department, Barcelona Supercomputing Center (BSC), Barcelona, Spain; d Centro de Investigacion Biomedica en Red Cancer (CIBERONC), Madrid, Spain; e Institucio Catalana de Recerca i Estudis Avançats (ICREA), Barcelona, Spain; f Physiological Sciences Department, School of Medicine and Health Sciences, University of Barcelona (UB), Barcelona, Spain

**Keywords:** Human, DNA methylation, microarray, epigenetics, CpG sites, validation

## Abstract

DNA methylation, one of the best characterized epigenetic marks in the human genome, plays a pivotal role in gene transcription regulation and other biological processes in humans. On top of that, the DNA methylome undergoes profound changes in cancer and other disorders. However, large-scale and population-based studies are limited by high costs and the need for considerable expertise in data analysis for whole-genome bisulphite-sequencing methodologies. Following the success of the EPIC DNA methylation microarray, the newly developed Infinium HumanMethylationEPIC version 2.0 (900K EPIC v2) is now available. This new array contains more than 900,000 CpG probes covering the human genome and excluding masked probes from the previous version. The 900K EPIC v2 microarray adds more than 200,000 probes covering extra DNA cis-regulatory regions such as enhancers, super-enhancers and CTCF binding regions. Herein, we have technically and biologically validated the new methylation array to show its high reproducibility and consistency among technical replicates and with DNA extracted from FFPE tissue. In addition, we have hybridized primary normal and tumoural tissues and cancer cell lines from different sources and tested the robustness of the 900K EPIC v2 microarray when analysing the different DNA methylation profiles. The validation highlights the improvements offered by the new array and demonstrates the versatility of this updated tool for characterizing the DNA methylome in human health and disease.

## Background

DNA methylation is one of the best studied epigenetic marks in the human genome. It consists of the covalent addition of a methyl group to the fifth carbon of a cytosine base (5-methylcytosine, 5mC) of the DNA. It is normally restricted to the context of cytosine and guanine dinucleotides (CpG). Certain regions of the genome are enriched in CpGs, forming CpG islands, which are regions of 200 bp to 2 kb in length, with a GC content greater than 50%, that are normally annotated in promoter regions [1,2]. From a functional point of view, DNA methylation alters chromatin structure and other epigenetic marks, such as histone modification, transcription factors, etc., which ultimately regulate gene expression [1,3]. DNA methylation is essential for regulating tissue-specific gene expression, X chromosome inactivation, genomic imprinting, and for inactivating repetitive genomic regions, among other functions [1]. Importantly, the influence of the DNA methylation may differ according to its genomic location. For example, methylation at promoter regions normally inhibits gene expression, but usually promotes it when it occurs in the gene body [1]. The DNA methylome is tightly regulated, so altered DNA methylation can cause various human disorders such as autoimmune, metabolic or neurological diseases and cancer [1,2]. Understanding the aberrant landscape of DNA methylation in human diseases is a challenging task, although important findings are emerging. For instance, it is widely known that human cancers feature a global loss of methylation relative to paired-normal tissue. Global hypomethylation can cause the reactivation of retrotransposons that are silenced in normal cells. In contrast, specific hypermethylation events in the promoter regions of tumour suppressor genes lead to the dysregulation of regulatory circuits of cell proliferation and homoeostasis [2]. Understanding the differential methylation patterns of the human DNA methylome between health and disease is of great clinical relevance since it can help identify useful biomarkers. Two examples are the methylation status of the *MGMT* gene, which may be used as a drug-sensitivity biomarker to predict the response to alkylating agents in glioma patients [4], and the methylation status of the *PPARγ* promoter, whose identification allows the stratification of liver fibrosis patients in non-alcoholic fatty liver disease [5]. DNA methylation is also very important as a target for new drugs currently in clinical use to reverse the aberrant methylation patterns of haematological malignances, such as DNA methyltransferase inhibitors (decitabine and azacytidine), in order to treat myeloid malignancies [6,7].

The necessity to understand fully the human DNA methylome in the context of human health and disease has been recognized since the development of the cutting-edge technology that allows DNA methylation to be measured, not only across large portions of the human genome but also at the population level. The current ‘gold standard’ technique is whole-genome bisulphite sequencing (WGBS), which provides single-nucleotide resolution and whole-genome resolution with 95% coverage of all CpGs in the human genome (≈28×10^6^ CpGs). However, the technique is costly and requires relatively large amounts of DNA. Along with the considerable technical expertise needed to process the data, these limitations make it a relatively impractical choice for population-based analyses [8,9]. More user-friendly DNA methylation arrays have been developed to overcome these disadvantages, but in exchange for offering more restricted genome coverage. Among these, Illumina have developed and improved several BeadChip human microarrays: the HumanMethylation27 BeadChip, which covers more than 27,000 CpGs (27K); the HumanMethylation450 BeadChip, which covers more than 450,000 CpGs (450K) [10]; and the HumanMethylationEPIC BeadChip, released in 2016, which covers more than 850,000 CpGs (EPIC array or 850K) [11]. In addition, given the large number of mouse models in translational research, a mouse DNA microarray has been developed that covers more than 285,000 CpG sites of the mouse genome [12]. These methylation microarrays have been widely used by the research community, not only for The Cancer Genome Atlas (TCGA) methylation studies [13], but also for deciphering the DNA methylome in genetic diseases [14], ageing [15], interindividual variability [16,17] and molecular epidemiology projects [18]. CpG probes map CpG-rich regions, gene promoters and cis-regulatory elements, mainly in the 850K array. However, while the coverage of these regulatory elements was extended by the development of the 850K array [11], there was still room for improvement. The growing knowledge about the impact of enhancers, super-enhancers and boundary elements, such as CTCF-binding domains, and their impact on transcription, means that a wider coverage of these distal transcriptional regulatory regions is needed. Several research studies have shown that the disruption of normal DNA methylation patterns of super-enhancers or CTCF-binding domains plays important roles during malignant transformation in cancer [3,19,20] and in the progression of neurodegenerative diseases [21,22]. Accordingly, a more complete knowledge of the role of DNA methylation in these regulatory regions is essential if our understanding of human disorders is to be improved. To meet these requirements Illumina has recently developed a new version of the Infinium HumanMethylationEPIC v2.0 BeadChip (900K) that contains more than 900,000 CpG probes, 77.63% of which are homologous with the first version, and more than 200,000 of which are new CpGs covering regions located in the open chromatin and enhancer regions identified by the ENCODE [23,24] and FANTOM5 [25] projects. Herein, we have validated the new 900K human methylation microarray from technical, functional and biological standpoints using cancer cell lines and normal and tumoural primary tissues. The results obtained demonstrate that the 900K microarray is a robust and reliable tool for interrogating the DNA methylome and thereby advancing our understanding of human DNA methylation mechanisms in development, ageing and disease, in particular regarding the landscape of cis-regulatory elements.

## Results and discussion

### Genomic characterization of the more than 900,000 cytosine sites in the new human 900K DNA methylation array

The MethylationEPIC BeadChip Infinium microarray v1.0 (850K) was initially developed in 2016 for the human GRCh37/hg19 [11], although the increasingly frequent use of the new version of the human genome GRCh38/h38 prompted the initial manifest to be updated to this latest version. Therefore, the 850K microarray in this study was characterized using the newest EPIC public manifest, which is available at https://zwdzwd.github.io/InfiniumAnnotation [26]. The 850K microarray interrogated the methylation status of 870,143 (865,918 unique) cytosine positions in the human genome covering the 22 autosomes, 1 sex chromosome pair and the M chromosome. The recently developed MethylationEPIC BeadChip Infinium microarray v2.0 (900K) annotated to the most recent version of the human genome (GRCh38/h38). It featured 936,866 (929,834 unique) CpG probes (900K microarray public manifest available at https://zwdzwd.github.io/InfiniumAnnotation), which covers all the chromosomes of the human genome that the 850K microarray does.

**Figure 1.**
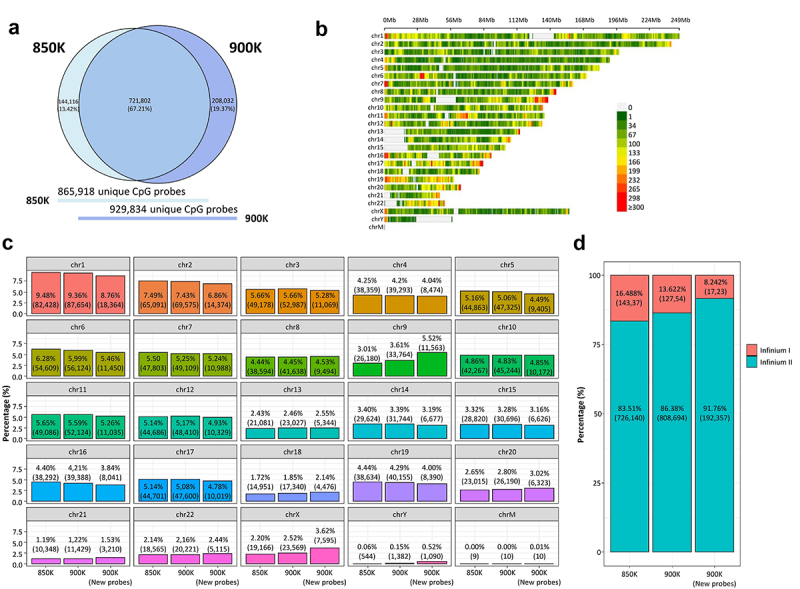
Comparison of the genomic context of the MethylationEPIC v1.0 (850K) and v2.0 (900K) BeadChip microarrays and the newly added CpG probes. (a) Venn diagram showing the degree of overlap (dark blue) between 850K (light blue) and 900K (deep-dark blue) microarrays. (**b**) CpG density plot of the 22 autosomes, 1 sex chromosome pair and M chromosome of the human genome showing the number of new CpGs in 900K microarray within 1-Mb-sized windows. The horizontal axis represents chromosome length (Mb) and the different colours indicate CpG density. (**c**) Bar plots represent the percentage of CpG for each chromosome pertaining to the 850K and 900K microarrays and the new probes of the 900K microarray. (**d**) Stacked bar plot represent the percentage of the Infinium design chemistry (Infinium I or Infinium II) of the probes in the 850K and 900K microarrays and the new probes of the 900K microarray.

Considering the CpG probes common to the 850K and 900K microarrays (Figure 1a), 726,597 were shared by the two arrays (721,802 unique probes, 67.21%). The complete list of shared probes is presented in Supplementary Table S1. 144,286 cytosine sites (144,116 unique) present in the 850K microarray were excluded from the new version of the array; these are listed in Supplementary Table S2. Remarkably, the 900K microarray accounts for 209,634 (208,032 unique) new CpG probes, listed in Supplementary Table S3, which increased the resolution of the human DNA methylome. Illumina methylation arrays also interrogated CpH sites (where ‘H’ corresponds to any nucleotide except guanine). 2,962 probes in the 850K microarray were situated within these sites, while the 900K microarray accounted for 2,914 probes. 2,785 probes were common to both arrays, while 129 probes were new to the 900K microarray (Supplementary Table S4). The new array reduces the number of SNP sites from 115 in the 850K microarray to 65 in the 900K version (Supplementary Table S5). The 209,634 extra CpG probes added in EPICv2 were distributed among all 22 autosome pairs, both sex chromosomes and the M chromosome. As may be seen in Figure 1b, these new probes increased the resolution of the 5’-starting positions of chromosomes 16 and 19, and the 3’-ending positions of chromosomes 9 and 17, as the density of CpG probes is higher in these regions. Overall, in the 900K microarray, chromosome 1 contained the greatest number of CpGs (*n* = 87,654, 9.36%) while chromosome 21 had the fewest positions (*n* = 11,429, 1.22%) for autosomal chromosomes, following the same trend as in the 850K microarray (Figure 1c). Interestingly, chromosome 9, which is not one of the longest chromosomes, is that with the second largest increase in the number of new CpG probes per chromosome (*n* = 11,563 new CpG probes, 5.52%), after chromosome 1 (*n* = 18,364 new CpG probes, 8.76%) (Figure 1c).

In contrast to the 850K microarray, the new 900K version increased the number of Infinium II probes design (808,694 and 726,140 CpG probes in the 900K and 850K microarrays, respectively), and decreased the number of Infinium I designed probes (127,537 and 143,368 CpG probes in the 900K and 850K microarrays, respectively) (Figure 1d). The design of the type II CpG probes not only helped to target low CpG-dense regions in the new array, but also reduced the physical space relative to the type I CpG probes, allowing an increased capacity for the CpG probes in the same chip space, as previously discussed [8].

### Functional characterization of the more than 900,000 cytosine sites in the new 900K human DNA methylation array

**Figure 2.**
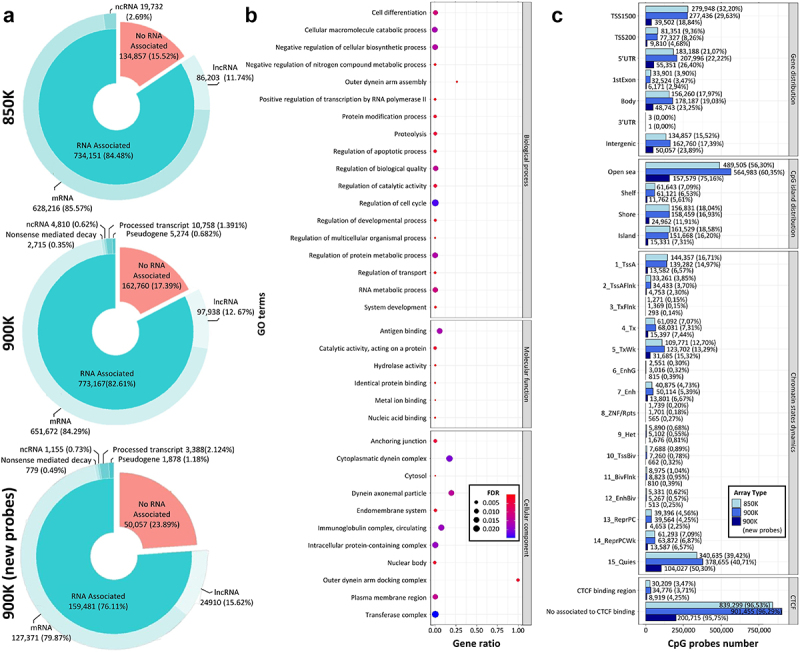
Comparison of the functional context of the MethylationEPIC v1.0 (850K) and v2.0 (900K) BeadChip microarrays and the newly added CpG probes. (**a**) Donut pie plot representing the percentage of CpG probes associated with RNA transcripts according to the GENCODE annotation in the 850K (top) and 900K (middle) microarrays and the new probes from the 900K (bottom) microarray. (**b**) Gene Ontology analysis of the additional transcripts in the 900K compared with the 850K microarray. The pathway name is indicated on the y-axis; the x-axis shows the ratio between observed and expected genes in a GO pathway. Bubble size and colour indicate the false-discovery rate (FDR): large blue and small red represent high and low values, respectively. (**c**) Bar plot representing the number of CpG probes in several annotation features of the microarray manifest: CpG probes in relation to gene (gene distribution) and CpG islands (CpG island distribution), and CpG probes associated with the different chromatin states model (chromatin state dynamics) and with CTCF-binding regions (CTCF-binding sites). Light, dark and deep-dark blue correspond to the 850K and 900K microarray probes and the new probes of the 900K microarray, respectively.

The new EPIC version was designed to increase the coverage of intergenic regions, so the association of the CpG probes with genes was assessed. In the new 900K microarray version, there were 50,153 new CpG probes interrogating these intergenic regions, adding a total of 162,760 (17.39%) CpG probes not associated with any type of RNA transcript. Moreover, comparing the probes associated with RNA transcripts in the 900K microarray revealed more probes to be associated with mRNA than with the 850K microarray (*n* = 651,672; 84.29% and *n* = 628,216; 85.57%, respectively). The same trend was observed for the transcript associated with lncRNAs, whereby the 900K microarray featured 97,938 (12.67%) CpG probes associated with these transcripts, while only 86,203 (11.74%) CpG probes were related to lncRNAs with the 850K microarray (Figure 2a). The functionality of new transcripts that were not present in the previous 850K microarray was then assessed. The 900K microarray targeted 16,481 more RNA transcripts than did the 850K microarray. To study this set of new transcripts covered by CpG probes in the 900K microarray further, functional gene annotation was carried out by gene set enrichment analysis (GSEA). The results, summarized in Figure 2b, showed an overrepresentation of Gene Ontology (GO) biological processes related to cell differentiation and regulation of metabolism; GO molecular functions such as antigen, protein, metal ion and nucleic acid binding; and an enrichment of GO cellular components associated with the endomembranous system, plasma membrane and nuclear body, among others.

We then annotated the CpG probes, in the 850K and 900K manifests, in relation to their location relative to the gene: in intergenic regions, the 1500 bp upstream transcription starting site (TSS1500), the 200 bp upstream transcription starting site (TSS200), 5’UTR, first exon (1stExon), gene body (body) or 3’UTR. This level of annotation, presented in Supplementary Tables 6 and 7, is not available in the public manifest. Following this functional classification, 63.58% (*n* = 595,283) of the CpG probes were located near proximal promoter regions (TSS1500, TSS200, 5’UTR or 1stExon) and 19.03% (*n* = 178,188) were associated with the gene body (body or 3’UTR). The same trend was seen in the 850K microarray annotation (Figure 2c). Surprisingly, the analysis of the genomic distribution of CpGs in relation to CpG islands revealed that the majority of the new CpG probes in the 900K microarray were in open sea regions (*n* = 157,579, 75.16% of new CpGs), increasing the total number of CpGs covering these regions to 564,983 (60.35%) compared with the 489,505 (56.30%) CpGs in the 850K microarray. Regarding CpGs associated with CpG islands, 16.20% (*n* = 151,668) were in CpG islands, 16.93% (*n* = 158,459) were in CpG island shores (sequences 2 kb upstream and downstream of the CpG island) and 6.53% (*n* = 61,121) were annotated in CpG island shelves (sequences 2 kb upstream and downstream of CpG island shores), following a similar pattern to that of the 850K microarray annotation (Figure 2c). We analysed the different chromatin states [27] associated with the CpG probes, taking advantage of the fact that this new DNA methylation array is designed to cover gene-regulation elements. The comparison of the 850K and 900K microarrays revealed the same pattern, except for the chromatin state associated with quiescence, in which 50.30% (*n* = 104,027) of the new 900K microarray probes were related (Figure 2c). Finally, the manifest annotation also characterized whether the CpG probes were associated with a CTCF-binding region. In this regard, the new microarray had 8,919 (4.25%) new CpGs associated with CTCF-binding regions for a total of 3.71% (*n* = 34,776) of the whole 900K microarray, while most of the newly added probes occurred in regions where CTCF was not bound (*n* = 200,715; 95.75%) (Figure 2c). Other detailed information about the CpGs included in the new EPIC array can be found at (https://zwdzwd.github.io/InfiniumAnnotation) [26] or in our manifest files (Supplementary Tables 6 and 7).

### Technical validation of the new 900K human DNA methylation array

**Figure 3.**
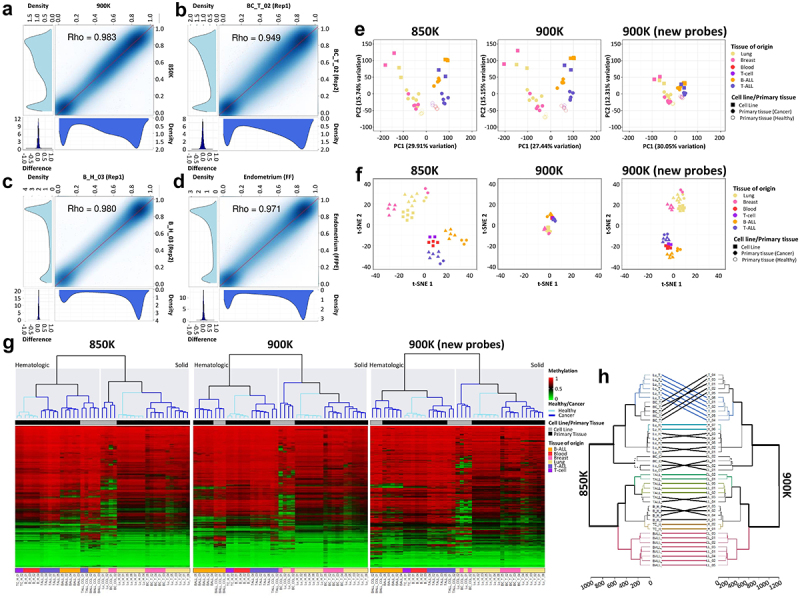
Technical and biological validation of the methylation profiles of the same samples hybridized in the MethylationEPIC v1.0 (850K) and v2.0 (900K) BeadChip microarrays. (**a-d**) Correlation plots of the CpG methylation values within (**a**) the same sample hybridized in the 850K and 900K microarrays; **(b**) technical replicate of the breast cancer primary sample; (**c**) technical replicate of the blood primary sample; and (**d**) consecutive fresh-frozen and formalin-fixed paraffin-embedded (FFPE) sections of normal endometrium primary tissue. At the bottom and to the left of each correlation graph, density plots show the methylation β-values corresponding to the sample on the x-axis (bottom plot) or the sample on the y-axis (left plot). At the bottom-left edge, density plots show the methylation differences between the two samples. (**e**) Biplot representing principal component (PC) 1 and PC2 of β-values of samples hybridized in the 850K (left) and 900K (middle) microarrays, and for the additional probes in the new 900K microarray (right). Colors represent the different tissues, dots indicate whether samples are primary tissue or cell line samples, and unfilled and filled dots represent healthy and tumoural tissue, respectively. (**f**) Biplot of the t-SNE analysis of samples hybridized in the 850K (left) and 900K (middle) microarrays and for the additional probes in the new 900K microarray (right). Colors represent the different tissues and dots indicate whether samples are primary tissue or cell line samples. (**g**) Unsupervised hierarchical analysis and heatmap for the 43 different samples hybridized in the 850K (left) and 900K (middle) microarrays and for the additional probes in the 900K (right) microarray. Colors indicate whether the sample is from primary tissue (normal or tumoural) and the tissue type, as described in the legend of the heatmap. Methylation β-values range from 0 (green) to 1 (red). (**h**) Dendrogram comparison of 43 samples hybridized in the 850K and 900K microarrays. Bold and coloured branches and lines between dendrograms indicate subtrees common to the two microarrays. Distinct edges in the 850K and 900K microarray dendrograms are shown as dashed branches.

We adopted a variety of approaches to demonstrate the reliability of the 900K microarray for analysing DNA methylation. First, we technically validated the array by hybridizing a lung tumour sample in the 900K methylation microarray, comparing the results obtained with the previously validated and standardized 850K microarray. The analysis of the probes common to both arrays demonstrated a close correlation between the methylation levels detected at each CpG site (Spearman correlation coefficient Rho = 0.983; *p* < 0.0001) (Figure 3a). A technical replicate validation was also performed by hybridizing the same sample (breast cancer and blood primary samples) twice in two different 900K microarray chips. As may be seen in Figure 3b,c the methylation values derived from each experiment were highly correlated (Spearman correlation coefficients: breast cancer sample, Rho = 0.949, *p* < 0.0001; blood sample, Rho = 0.980; *p* < 0.0001). Next, as the 900K microarray is compatible with DNA extracted from formalin-fixed, paraffin-embedded (FFPE) tissue samples, we hybridized two samples from normal human endometrium primary tissue obtained from consecutive fresh or FFPE sections to test the robustness of the assay. The results demonstrated the optimal performance of the 900K microarray for FFPE samples, considering the close correlation of each CpG site between fresh and FFPE samples (Spearman correlation coefficient Rho = 0.971, *p* < 0.0001) (Figure 3d). Considering that we were hybridizing primary tissue for this experiment, it is important to consider cellular heterogeneity as a potential limitation. However, as the FFPE section was close to the fresh-frozen section, significant differences in cellular heterogeneity were discounted [11,12]. Taken together, our results confirmed the robustness and reproducibility of the new 900K microarray in fresh-frozen and FFPE tissue.

### Biological validation of the new 900K human DNA methylation array

Given that DNA methylation is tissue-specific, we examined the feasibility and reproducibility of the 900K microarray to assess the different DNA methylation profiles of 43 different samples in comparison with the previously validated 850K microarray [11]. Normal and tumoural primary tissues and cancer cell lines from haematological malignancies (T-cell acute lymphoblastic leukaemia, T-ALL, and B-cell acute lymphoblastic leukaemia, B-ALL) and solid tumours (lung and breast) were hybridized in 850K and 900K microarrays. A detailed list of the samples can be found in Table 1. Three types of unsupervised analysis were done. First, we performed a principal component analysis (PCA), which uses a type of unsupervised linear dimensionality reduction to explore the DNA methylation profile of the various samples. We observed that, in both the 850K and 900K microarrays, samples clustered together depending on whether they were from haematological or solid tissue. Slight differences were found between 850K and 900K microarray PCAs. Interestingly, by using only the new probes added in 900K microarray, samples still clustered on the basis of their tissue of origin (haematological or solid tissue) (Figure 3e). Similar results were obtained with the t-SNE analysis, a type of unsupervised non-linear dimensionality reduction, whereby the haematological and solid samples clustered together. The 900K microarray data preserved the local structure of the data better, distinguishing the two clusters more clearly (Figure 3f). As before, the 900K-specific probes were useful for representing the biological characteristics of the samples (Figure 3f). An unsupervised hierarchical clustering approach using random CpGs was performed. Consistent with the PCA and t-SNE analyses, the DNA methylation profiles of the samples were significantly different, to the extent that samples of haematological origin clustered separately from samples of solid origin in the 850K and 900K microarrays. Considering the findings from both microarrays in greater detail, within haematological and solid clusters, samples clustered with respect to whether they were normal or tumoural tissues (Figure 3g). It was notable that, while all cancer cell lines clustered independently of their tissue of origin in the 850K microarray, cell lines clustered within the same haematological tissue in the 900K microarray (Figure 3g). Furthermore, the unsupervised hierarchical analysis of the new CpG probes of the 900K microarray was able to cluster samples according to whether they came from haematological or solid tissue, and, within each cluster, if they were from normal or tumoural tissue. The haematological samples clustered together with respect to their sample type and tissue of origin (Figure 3g). We performed a further unsupervised clustering analysis that included all possible CpGs and compared the resulting dendrograms. As may be seen in Figure 3h, most of the branches were common to both dendrograms, with few samples differentially located within the same clade. Taken together, these results demonstrated the great robustness of the new 900K methylation microarray compared with the 850K microarray.

**Table 1.** List of samples used for the technical and biological validation of the Human MethylationEPIC v2.0 microarray.

Platform	Hematological/Solid	Tissue of origin	Tissue type	n
850K (*n* = 43)	Haematological (*n* = 21)	Blood	Normal tissue	4
		T-cells	Normal tissue	2
		T-ALL	Cancer cell lines	2
			Tumour tissue	5
		B-ALL	Cancer cell lines	3
			Tumour tissue	5
	Solid (*n* = 22)	Lung	Normal tissue	7
			Cancer cell lines	2
			Tumour tissue	6
		Breast	Cancer cell lines	2
			Tumour tissue	5
900K (*n* = 47)	Haematological (*n* = 22)	Blood	Normal tissue	4 (+1 rep)
		T-cells	Normal tissue	2
		T-ALL	Cancer cell lines	2
			Tumour tissue	5
		B-ALL	Cancer cell lines	3
			Tumour tissue	5
	Solid (*n* = 25)	Lung	Normal tissue	7
			Cancer cell lines	2
			Tumour tissue	6
		Breast	Cancer cell lines	2
			Tumour tissue	5 (+1 rep)
		Endometrium	Normal tissue	2 (FF + FFPE)

Abbreviations: B-cell acute lymphoblastic leukaemia (B-ALL); T-cell acute lymphoblastic leukaemia (T-ALL); MethylationEPIC v1.0 (850K); MethylationEPIC v2.0 (900K).

**Figure 4.**
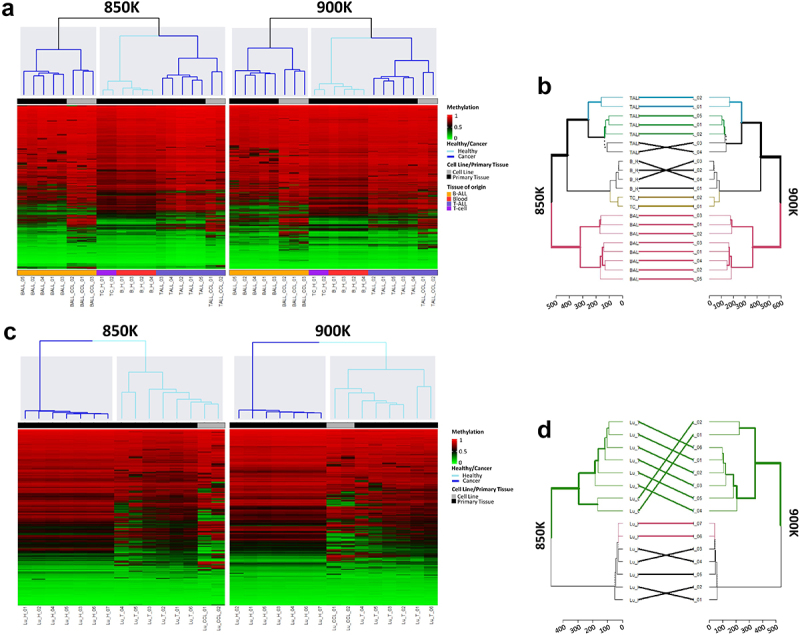
Unsupervised hierarchical analysis of methylation profiles of blood and lung sample revealed by the MethylationEPIC v1.0 (850K) and v2.0 (900K) BeadChip microarrays. (**a**) Unsupervised hierarchical analysis and heatmap from the 21 haematological samples hybridized in the 850K (left) and 900K (right) microarrays. Colors indicate whether the sample is from primary tissue (normal or tumoural) and the tissue type, as described in the legend of the heatmap. Methylation β-values range from 0 (green) to 1 (red). (**b**) Dendrogram comparison of 21 haematological samples hybridized in the 850K and 900K microarrays. Bold and coloured branches and lines between dendrograms indicate subtrees common to the two microarrays. Distinct edges to the 850K and 900K microarray dendrograms are shown as dashed branches. (**c**) Unsupervised hierarchical analysis and heatmap from the 15 lung samples hybridized in the 850K (left) and 900K (right) microarrays. Colors indicate whether the sample is from primary tissue (normal or tumoural) and the tissue type, as described in the legend of the heatmap. Methylation β-values range from 0 (green) to 1 (red). (**d**) Dendrogram comparison of 15 lung samples hybridized in the 850K and 900K microarrays. Bold and coloured branches and lines between dendrograms indicate subtrees common to the two microarrays. Distinct edges to the 850K and 900K microarray dendrograms are shown as dashed branches.

Finally, the experimental design enabled us to perform unsupervised hierarchical analyses of normal and tumoural tissue from lung and blood. For the blood samples, the analysis using random CpGs revealed distinct methylation profiles between the B-ALL samples and all the other haematological samples. It was possible to distinguish between healthy and tumoural haematological samples (Figure 4a). The resulting dendrograms from the unsupervised hierarchical analysis with all the CpGs from the 850 and 900K microarrays were nearly identical (Figure 4b). In the case of the lung samples, distinct methylation profiles were clearly identified between normal and tumoural tissues in the 850K and 900K microarrays (Figure 4c), while the branches of the dendrograms arising from the unsupervised hierarchical analysis of all the CpGs from the 850K and 900K microarrays differed only slightly (Figure 4d).

Taken together, these results highlight the biological reproducibility and feasibility of the new 900K methylation microarray. We have demonstrated that the 900K methylation array reliably detects the distinct DNA methylation profiles of normal and tumoural primary samples and cancer cell lines, as does the 850K microarray. The 900K microarray proves to be a promising and interesting tool for further interrogating the cis-regulatory regions of the human genome at higher resolution than is possible with the 850K microarray.

## Conclusions

Our study presents a technical and biological validation of the new Illumina HumanMethylationEPIC v2.0 microarray. We used it to validate the methylation status of 936,866 CpG probes in human normal and tumoural primary samples and in human cancer cell lines. The new 900K microarray represents a significant improvement on the 850K microarray, adding more probes in intergenic and open sea regions with which the methylation status of the DNA cis-regulatory elements may be interrogated. Technically, the 900K microarray proved the reproducibility to assess the DNA patterns between technical replicates and also in FFPE tissue samples. Finally, our biological validation confirmed that the new microarray can distinguish DNA methylation patterns between samples, thereby enabling clustering according to tissue type, and normal compared with tumoural states. Overall, the new 900K microarray ensures that methylation arrays remain a low-cost tool with which to examine a relatively large proportion of the human DNA methylome using a population-based approach with relatively simple bioinformatic analyses.

## Materials and methods

### Samples

All 45 samples examined in this study were of human origin. They included human cancer cell lines (breast, lung, T-ALL and B-ALL), normal primary tissues (lung, endometrial, blood and T-cells) and tumoural primary tissues (breast, lung, T-ALL and B-ALL). The tissues were either fresh-frozen or formalin-fixed, paraffin-embedded (FFPE). All samples were obtained following approval by the Institutional Ethics Committee.

### DNA extraction

DNA isolation from FFPE embedded samples was performed using the E.Z.N.A.® FFPE DNA Kit (Omega Bio-Tek USA). In the case of fresh-frozen samples the, DNeasy Blood & Tissue Kit (Qiagen, Hilden, Germany) was used following the manufacturer’s instructions. All DNA samples were treated with RNase A for 1 h at 45ºC, and DNA quantities were assessed using the Quant-iT PicoGreen dsDNA Assay (Life Technologies, CA, USA). DNA purity was determined with NanoDrop (Thermo Scientific, MA, USA) by measuring the 260/280 and 260/230 ratios. DNA integrity of the samples was checked by electrophoresis.

### Quality check of FFPE DNAs

Following Illumina’s recommendations, the DNA quality of the FFPE tissues was checked by performing a quantitative PCR with 2 ng of FFPE DNA. The average value of a standard provided by Illumina was subtracted in order to calculate the ΔC_q_. FFPE DNAs were shown to be properly restored provided their ΔC_q_ of<5, indicating adequate FFPE restoration.

### Bisulphite conversion

DNA samples were distributed on a 96-well plate in a randomized manner. DNA concentrations used were as recommended by the manufacturer (300ng and 600ng for FFPE and fresh-frozen tissue respectively). EZ-96 DNA Methylation kit (Zymo Research Corp., CA, USA) was then used to process samples according to the fabricant’s instructions.

### FFPE restoration

As previously described by Moran *et al*. [28] in order to restore bisulphite-converted DNA from FFPE, DNA was denatured with NaOH for 10 min at room temperature. PPR (Prime Pre Restore) reagent and Amplification Mix Restore reagent were then added and samples were incubated for 1 h at 37ºC. DNA was washed with ZR-96 DNA Clean & Concentrator-5 kit (Zymo Research Corp.) and eluted in 13 µl of ERB, followed by a denaturation step (2 min incubation at 95ºC) and ligation (1 h incubation with RST and CMM reagents). Finally, DNA was washed again with ZR-96 DNA Clean & Concentrator-5 kit (Zymo Research Corp.) and eluted in 10 µl of DiH_2_O.

### Array hybridization

The MethylationEPIC v2.0 BeadChip shares the Infinium HD chemistry Assay (Illumina Inc.) used for the MethylationEPIC v1.0 BeadChip. Therefore, the appropriate protocol for the MethylationEPIC v2.0 is the same as that used for the MethylationEPIC v1.0 Assay Protocol: the Infinium HD Methylation Assay Protocol. Therefore, 8 µl or 4 µl of bisulphite-converted DNA from FFPE or fresh-frozen samples, respectively, were used for processing. The protocol used was as described elsewhere [10,11].

### MethylationEPIC versions 1.0 and 2.0 manifests

The most recent versions of the human CpG annotation manifests for MethylationEPIC v1.0 and v2.0 were downloaded from http://zwdzwd.github.io/InfiniumAnnotation [26]. Files regarding basic mapping information, mask information, gene annotation (GENCODEv41 and GENCODEv36 for microarray v1.0 and v2.0, respectively), CpG island annotation and SeSAMe annotations were downloaded and merged in a single file for each microarray version. We also added the annotation of CpG in relation to genes. For this purpose, GENCODE v36 (850K microarray) and GENCODE v41 (900K microarray) were downloaded from the GENCODE data portal. CpGs associated with transcripts were annotated with respect to whether the CpG was located in transcription starting site regions, defined as ± 1500 bp around transcript start sites (TSS1500) or ± 200 bp around transcript start sites (TSS200). The 5’UTR region was defined as the region between the start of the transcript and of the first exon. The latter was defined as the region between the start and end of the first exon. The aforementioned regions were considered as promoter regions, while gene body regions were considered regions between the ends of the first exon and of the last transcript exon (body) and the 3’UTR regions between the ends of the last transcript exon and of the transcript. CpG probes associated with intergenic regions were defined as CpGs annotated in regions of the genome that did not overlap with the body or with promoter regions. The complete manifests used in this article can be found in Supplementary Tables 6 and 7.

### Marker classification

CpG probes were classified by their chromosomal location, and the Infinium design was used to interrogate the CpG region in the genome (Infinium I or Infinium II), the location of the CpG in relation to its CpG context (CpG island, shore, shelf, open sea) and in relation to the gene region (TSS1500, TSS200, 5’UTR, first exon, body, 3’UTR or intergenic). As some CpG probes were annotated in multiple gene and TSS locations, categories were prioritized according to a 5’-prime to 3’-prime criterion (TSS1500 > TSS200 > 5’UTR > first exon > body>3’UTR > intergenic). Likewise, CpG annotation was carried out in relation to CpG islands, so that the prioritization was as follows: CpG island > CpG island shore > CpG island shelf > open sea. Other criteria included the location of the CpG in relation to chromatin states according to the 15 chromatin states model [27]. The association of CpGs with CTCF-binding domains [29] was also interrogated.

### Functional gene annotation

Transcripts associated with CpG probes in the MethylationEPIC v1.0 and v2.0 arrays were compared, and the new transcripts annotated in the MethylationEPIC v2.0 array were tested in a functional gene annotation by gene set enrichment analysis (GSEA) using the gene ontology (GO) online tool (http://geneontology.org) [30]. GO biological process, molecular function and cellular component annotation datasets were assessed with Fisher’s exact test and corrected for the false discovery rate (FDR). Only GO pathways with an FDR <0.05 were considered significant.

### Data normalization and statistics

The resulting raw signal intensities (IDATs) were assessed and analysed using GenomeStudio 2011.1 (Illumina). DNA methylation beta values were obtained after GenomeStudio default normalization using control probes and background subtraction. Several quality and control steps were performed with the aim of minimizing errors and removing erratic probe signals. This involved removing probes with a detection value of *p* > 0.01, XY chromosomes probes, genotyping probes for the DNA methylation studies and masked probes. Genomic analysis used the human genome hg38, as described in the manifest file associated with MethylationEPIC BeadChip versions 1.0 and 2.0.

### Unsupervised and other computational analyses

DNA methylation patterns from samples obtained from both microarray platforms were examined with different types of unsupervised analyses, all of which used a variety of R packages. Principal component analysis (PCA) was performed using the PCAtools package (v2.6.0). t-distributed stochastic neighbour-embedding (t-SNE) analysis was performed using the Rtsne package (v0.16). Two types of unsupervised hierarchical analyses were done, using random CpGs, where 1% or 100% of the CpGs from the samples were selected and analysed. In both cases, sample clustering was performed with 100 bootstrap replications, using Euclidean distances and the Ward.D clustering method available in the pvclust package (v2.2–0). Results were presented as a heatmap using ComplexHeatmap (v2.10.0) package, or as a dendrogram using the dendextend (v1.16.0) package. Density plots were performed using minfi (v1.40.0), correlation plots were designed using the smoothScatter function in graphics (v4.1.2), Venn diagrams were produced with VennDiagram (v1.7.3), CpG density plots were designed using CMplot (4.2.0), pie donut plots were produced with webr (v0.1.5) and bar plots were designed using ggplot2 (3.4.0).

## Supplementary Material

Supplemental Material

## Data Availability

The complete DNA methylation data from 900K EPIC V2 are freely available on the GEO repository under accession number GSE222919 (https://www.ncbi.nlbm.nih.gov/geo/query/acc.cgi?acc=GSE222919).
